# Quality of Work Life Determinants of Healthcare Professionals’ Quiet Quitting: Towards Individual Difference

**DOI:** 10.3390/healthcare13131547

**Published:** 2025-06-28

**Authors:** Milica Stankovic, Marko Slavkovic

**Affiliations:** 1 General Hospital “Sveti Luka”, Knez Mihaila 51, 11300 Smederevo, Serbia; milica.stankovic@obsmederevo.rs; 2 Department of Management and Business Administration, Faculty of Economics, University of Kragujevac, Liceja Knezevine Srbije 3, 34000 Kragujevac, Serbia

**Keywords:** quality of work life, quiet quitting, work-related outcomes, healthcare professionals, gender disparities in healthcare

## Abstract

**Background/Objectives:** Quality of work life (QWL) in the healthcare industry emerges as an important factor for enhancing positive and preventing negative work-related outcomes, including quiet quitting. The aim of the study was to investigate the impact of quality of work life on the indication of quiet quitting among healthcare professionals. **Methods:** A cross-sectional study design and convenience sampling method were applied. A minimum sample was estimated by applying Cochran’s formula with a 5% significance level and 95% confidence interval. The target population of the study consisted of healthcare professionals employed in public health organizations in central Serbia, with a total sample size of 647 respondents. Testing the relationship between determinants of quality of work life and quiet quitting was conducted through a structural equation modeling approach based on partial least squares (PLS-SEM). **Results:** The results indicate that psychological, physical, and cultural quality of work life have a significant impact on the manifestation of quiet quitting among healthcare professionals, especially among women. **Conclusions:** Findings suggest that social well-being is significant only for men in relation to quiet quitting. The findings reveal the elements of quality of work life are associated with the occurrence of quiet quitting among healthcare professionals, thus serving as a solid starting point for formulating effective human resource management strategies that can prevent negative consequences.

## 1. Introduction

In recent decades, due to the process of globalization, technological innovations, and changes in labor requirements, social and economic transformations have occurred that have placed additional demands on employees and influenced people to change their attitude towards work [1,2]. Employees tend to meet their needs in terms of health, economy, safety, social life, improvement of knowledge, and respect through work, and thus experience a higher level of quality of work life [3]. Quality of work life is a concept developed to identify the physical and psychological consequences that are manifested in an employee that are related to work [4,5]. It is defined as the degree to which work in an organization contributes to the needs of individuals being met, arising from the individual experiences of employees [6]. Some of the dimensions used to measure and evaluate the quality of work life are work environment, job security, job satisfaction, professional development opportunities, organizational culture, employee recognition, organizational and interpersonal relationships [7]. When employees are assigned to jobs that are in accordance with their abilities and personal characteristics, it increases the likelihood that employees will exhibit a higher level of job satisfaction and accomplishment, and this can contribute to employees perceiving a higher level of quality of work life [8]. A prominent level of quality of work life for employees can contribute to higher satisfaction, engagement, and productivity, as well as reduced rates of absenteeism and turnover, all of which can have a positive impact on the efficiency and effectiveness of an organization [7].

Nowadays, at the international level in various professions, including healthcare, quality of work life has become an important concept [9,10]. For a healthcare organization to achieve its mission of providing quality and safe services, qualified healthcare professionals are one of the most important resources [11]. In healthcare organizations, quality of work life is a significant variable of organizational behavior that can have an impact on work performance [12] and employee retention [13]. For a health organization to attract and retain qualified health professionals, it needs to provide a safe, fair, and flexible working environment, which can contribute to a higher level of quality of work life among employees [13]. Research shows that a low level of quality of work life contributes to reduced employee engagement, increased turnover, and reduced work performance [4], which can potentially contribute to a decrease in the quality of health services provided [14], and at the same time a decrease in customer satisfaction. For a high level of quality of work life and reduced turnover of health professionals, it is necessary to have the support of superiors in the workplace [15,16], the possibility of education, training, and advancement in the organization [17,18], and the introduction of flexible work engagements.

The work environment has a significant impact on the professional behavior of employees in the organization [19]. When a positive work environment prevails in an organization, employees can exhibit a higher level of work engagement during the performance of work tasks through greater dedication, energy, and absorption, complete concentration on work, imperceptible passage of working time, and more difficult separation from work tasks [19,20]. On the other hand, in recent years, the term quiet quitting has been used more often in the literature [21], which does not mean that the employee literally leaves their profession, but represents adopted patterns of professional behavior [22] where employees without dedicated work perform only work tasks that are in the description of the position to which they are assigned and do not perform any tasks that are outside the scope of work tasks [23,24]. In practical terms, employees have lost the will to work and give their minimum when performing work tasks, so they are often disinterested and disengaged [25,26].

Quiet quitting is not a novel concept, despite its significant success in previous years, primarily because it accurately reflects the core reality of the cutting-edge relationship between employers and employees. Thus, it is well founded and confirmed by existing theories. Social exchange theory explains quiet quitting as a breakdown in the workplace “give-and-take.” Rooted in the cost–benefit logic, the theory holds that employees stay engaged when they believe the rewards outweigh the costs of effort. Perceived organizational support is essential to this calculation: when employees perceive that their employer prioritizes their well-being, they respond with increased commitment, citizenship behavior, and performance. Conversely, when support is inadequate, they withdraw psychologically, reduce discretionary effort, or quietly quit [27]. According to the human need theory, all behavior is derived from the pursuit of universal needs. Consequently, when a workplace assists employees in fulfilling these needs, it enhances their well-being, energy, engagement, and intention to stay. In contrast, when needs are not met, the resulting loss of commitment and belonging leads to stress, disengagement, and, in the end, quiet quitting [21].

Quiet quitting is a term that is becoming increasingly prevalent in research related to the younger population of people [28]. However, it is present in all age populations and professions and is of particular concern in health [23] due to its potential negative impact on public health. Healthcare professionals face increasing demands for the provision of health services and demands for the health organization to increase its credibility [29], while on the other hand, the health organization does not provide the necessary resources, and employees lack the support of superiors and endure poor cooperation [30]. As a result, healthcare professionals experience dissatisfaction, burnout, increased levels of stress, and depression [29,31], which is why employees are more likely to opt for quiet quitting. Resistance on the part of healthcare professionals is due to the feeling that work overload endangers their family and disrupts social life and leisure time [32]. When healthcare professionals feel exhausted due to working with limited resources, and the health organization does not contribute to improving working conditions, employees decide to take certain measures so that the working environment does not disrupt the work–life balance and opt for quiet quitting [33]. In addition to quiet quitting, one of the significant problems faced by healthcare organizations due to employee dissatisfaction is high turnover [34]. Quite quitting in healthcare professionals is a reaction to unsatisfactory working conditions, and employees who intend to leave the profession are more likely to use quiet quitting as a transitional solution until they find a new suitable job [34]. A healthcare organization that is driven to be competitive and attract and retain qualified workers should focus its managerial activities on promoting positive mental states and well-being in employees. In this way, employees exhibit a higher level of quality of work life, greater engagement, and dedication to work, and at the same time reduce quiet quitting and fluctuation [35,36].

Quality of work life is a person’s emotional reaction to their work and has a significant impact on professional burnout and quiet quitting [23,37]. For the sustainability of the health system and building a healthier population, it is invaluable to invest in existing healthcare workers [38]. For employees to be motivated and dedicated to their work, job satisfaction is essential [39]. Healthcare professionals who are more satisfied provide services efficiently and effectively, give their best, have innovative ideas, and thus contribute to the improvement of the working environment, but also to the well-being of public health [40]. A key characteristic of a competitive healthcare organization is that it has a prevalent positive work environment [39,41], and thus, the organization can expect healthcare professionals to exhibit a lower level of quiet quitting [25]. On the other hand, there are a number of factors that lead to job dissatisfaction, some of which are work overload, stress, conflicts with superiors or colleagues, professional burnout, shift work, etc. [42], as a result of there being a greater possibility of quiet quitting in employees. In considering all of the above, increasing job satisfaction and preventing quiet quitting require a working environment in a healthcare organization that promotes the preservation of the physical, social, and mental health of employees.

In recent articles, quiet quitting is recognized as a significant risk for global healthcare [22] and alarming threat for healthcare organizations and services [43]. Numerous studies have investigated factors that cause healthcare professionals to quietly quit, such as job burnout [44,45,46], emotional intelligence [47], employee well-being [45], workplace bullying [29], innovation support [48], and organizational support [49]. Upon reviewing the available studies, it is evident that there are no studies that specifically examine the relationship between quality of work life and quiet quitting in healthcare settings. These clearly pose a research gap we are trying to overcome with this study. Consequently, the purpose of this study is to assess the importance of the relationship between psychological, physical, social, and cultural quality of work life and quiet quitting in healthcare professionals. Based on the above, the aim of this study is to investigate the impact of quality of work life on the manifestation of quiet quitting in healthcare professionals.

## 2. Materials and Methods

### 2.1. Participants and Procedure

Healthcare professionals are acknowledged as a key source of information for collecting primary data. For the purposes of this research, we decided to apply a cross-sectional study. The target population of this study was healthcare professionals employed in public health organizations in central Serbia. Prior to participation, we applied Cochran’s sample-size formula [50] to determine the required number of participants, with the most commonly applied significance level of 5% and a confidence interval of 95%. Based on the Health Statistical Yearbook of the Republic of Serbia [51], we found that the total population of healthcare professionals is 110,929. Consequently, we determined that the appropriate sample for this study should consist of a minimum of 383 respondents. The sample was formed according to the principle of a convenient sample, i.e., all employees of the surveyed health organizations who met the criteria and wanted to participate in the research were included in the research process itself. Although convenience sampling limits statistical generalizability, bias was mitigated by recruiting across multiple public health organizations on different weekdays, thereby widening the demographic spread of the sample. Each participating healthcare professional was asked to refer others who meet the study’s criteria, thereby recruiting additional participants through snowball referrals. To ensure compliance with ethical standards during sampling, the research conducted is in accordance with the latest amendments to the Declaration of Helsinki. For the implementation of this study, the approval of the Council of the Faculty of Medical Sciences of the University of Kragujevac was obtained (protocol number 01-8491 from 25 August 2023). The questionnaires were distributed in writing and were designed in such a way that on the first page there was a statement of informed written consent. All potential participants were given information about the purpose of the study, and before filling out the questionnaire, they were informed of how anonymity and data security would be ensured. There were no questions in the questionnaire relating to personal data that are sensitive in nature. Participants were given autonomy to decide whether to participate in the study and were informed that they could ask additional questions about the questionnaire or opt out of completing the questionnaire at any time. The study was conducted from June to September 2024, and the total sample consisted of 647 participants, greatly exceeding the required minimum of respondents.

### 2.2. Measurements

A structured questionnaire was used to collect the data necessary to conduct the intended statistical analysis. The questionnaire was designed in such a way that it consists of three parts. In the first part of the questionnaire, there were sixteen statements related to the measurement of the quality of work life of employees. In this study, the quality of work life is an independent variable and is measured through four dimensions: psychological, physical, social, and cultural quality of work life [52]. The second part of the questionnaire focuses on measuring the level of quiet quitting in healthcare professionals. In this study, quiet quitting was viewed as a dependent variable and was measured using the quiet quitting scale (QQS) [25]. This part of the questionnaire consists of statements such as “I do the basic or minimum amount of work without going above and beyond”, “How often do you take initiative at your work?”, and “How often do you pretend to be working in order to avoid another task?” All findings were translated from English into Serbian and adapted to the purposes of this study. Participants expressed their opinion on a five-point Likert scale, where a value of 1 refers to complete disagreement and a value of 5 refers to complete agreement with the stated items. Another 5-point Likert scale was utilized for certain statements, with 1 indicating “never” and 5 referring to “always”. The third part of the questionnaire aimed to collect information about the characteristics of the participants, such as gender and age.

### 2.3. Data Analysis

In this study, testing was conducted using the partial least squares (PLS-SEM) structural equation modeling approach performed in SmartPLS 4 statistical software. Confirmatory factor analysis (CFA), average extracted variance (AVE), and composite reliability (CR) were used to assess convergent validity and reliability. After the estimation of convergent validity, an estimation of discriminant validity was performed using the heterotrait–monotrait criterion (HTMT0.85). To evaluate the structural model, the PLS algorithm was used, which represents a cross-validated test of predictive relevance. Bootstrapping was performed as a standard procedure of PLS-SEM to assess the importance of relationships between constructions. Finally, we performed a comparative analysis of the path coefficients between diverse groups, which we categorized based on gender and age. The sample size of 647 subjects is significantly above the recommended minimum limit [53], which is why the data are considered suitable for PLS-SEM computation.

## 3. Results

### 3.1. Respondents’ Profile

In our study, the total sample size was 647 healthcare professionals. Females predominate in the sample (85.16%). Among the participants, 44.51% belonged to the age population up to 40 years, while 27.98% of the employed participants were over 51 years of age and 27.51% belonged to the age population of 41 to 50 years.

### 3.2. Measurement Model Assessment

To assess the reliability and validity of the proposed findings, a confirmatory factor analysis was applied. Table 1 shows the results of both internal consistency analyses and convergent validity analyses for the model. Cronbach’s alpha coefficient reached the threshold set for all latent variables [54]. Composite reliability (CR) in all measured constructions in our study exceeded the proposed limit of 0.7 [55], which revealed that there is a strong internal consistency of the data. Convergent validity was measured over the Average Variance Extracted (AVE) and is considered satisfactory when it exceeds a limit of 0.5, which means that it makes up for more than 50% of the variance in its items [56]. The AVE values in our study ranged from 0.524 to 0.810, indicating that the necessary criteria were met. The values of the variance inflation factor (VIF) suggest that multicollinearity in our study is not a concern, given that all VIF values were lower than five [57]. Item “How often do you pretend to be working in order to avoid another task?” listed in the quiet quitting scale did not meet the criteria of the confirmatory factor analysis, and it was excluded from further calculations.

In the assessment of discriminant validity, the heterotrait–monotrait method (HTMT0.85) was performed, and the results obtained are presented in Table 2. The maximum value of discriminant validity is 0.85 [58], and in our study, the highest value obtained is 0.798, while other values are lower. On this basis, it is confirmed that our measurement model has successful discriminant validity.

### 3.3. Structural Model Assessment

The assessment of the structural model started with the blindfolding procedure within the PLS algorithm. A cross-validated redundancy index (Stone-Geisser Q^2^) was calculated for a latent variable to assess the predictive relevance of the internal model. One of the rules proposed in the literature is that Stone–Geisser Q^2^ values greater than 0.00 indicate that model has a low accuracy and predictive relevance; values greater than 0.25 and 0.50 indicate medium and high accuracy and predictive relevance [59]. In our study, the calculated value of Stone–Geisser Q^2^ for quiet quitting is 0.462 (Table 3). Given that the Q^2^ values are positive and the quality of the structural model is quite high, the results obtained are considered acceptable. The analyzed coefficient of determination (R^2^) indicates that 47.4% of quiet quitting is explained by the model and contributes significantly to the explanatory power of the model. In addition, it was observed that the goodness-of-fit (GOF) value of 0.467 is within the acceptable range, since the ideal value should be between 0 and 1. The limit value of the standardized mean square residue (SRMR) is 0.08 [60]. In our study, the SRMR value is lower than the recommended value (0.073), which indicates that the model is well fitted.

The standard bootstrapping procedure in PLS-SEM has been used to estimate the importance and size of the path coefficients. This technique involves calculating a bilaterally corrected 95 percent confidence interval (CI) for all relationships listed in Table 4 and estimating the direct effects. Table 4 shows the results of the structural model, with all the lower and upper values of the CI, as well as the testing of direct effects. Statistical analysis revealed a negative and statistically significant relationship between psychological quality of work life (QWL-Ps) and quiet quitting (QQ) (β = −0.307; *p* < 0.001). Similarly, the relationship between physical quality of work life (QWL-Ph) and quiet quitting (QQ) was also negative and statistically significant (β = −0.136; *p* < 0.01). Contrary to previous results, the relationship between social quality of work life (QWL-S) and quiet quitting (QQ) was negative and had no statistical significance (β = −0.100; *p* > 0.05). Cultural quality of work life (QWL-C) has a statistically significant and negative effect on quiet quitting (QQ) (β = −0.247; *p* < 0.001). The supported negative relationships between QWL-Ps and QQ and between QWL-C and QQ lead to the finding that psychological and cultural quality of work life have a significant impact on the manifestation of quiet quitting in healthcare professionals.

In the next step, we conducted a comparative analysis of the path coefficient among diverse groups, which we grouped according to gender and age. The respondents were divided according to gender, forming two groups, while the grouping of participants according to age formed three groups. Regarding the effect of psychological quality of work life on quiet quitting, statistically significant results were observed in both women and men, with a negative impact observed in women (β = −0.308; *p* < 0.001). Statistically significant results were also observed in both sexes and in the impact of cultural quality of work life on quiet quitting. As in the previous case, a slightly stronger effect was observed in women (β = −0.233; *p* < 0.001). In looking at the effect of physical quality of work life on quiet quitting, it was found that it is statistically significant only in women (β = −0.174, *p* < 0.01). In contrast, the effect of social quality of work life on quiet quitting was statistically significant only in men (β = −0.299, *p* < 0.01). The results suggest that gender moderates the effects between all the observed dimensions of quality of work life and quiet quitting. When analyzing the impact of the age of healthcare professionals, a statistically significant difference was not observed between the social quality of work life and quiet quitting. When examining the impact of cultural quality of work life on quiet quitting, a statistically significant difference was observed in all age groups. More precisely, slightly more significant results were observed in subjects belonging to the age population from 41 to 50 years (β = −0.322, *p* < 0.001), and then in subjects who are less than 40 years old (β = −0.226, *p* < 0.01), and in subjects over 51 years of age (β = −0.187, *p* < 0.05). The impact of psychological quality of work life on quiet quitting was statistically significant for participants under 40 years of age (β = −0.336, *p* < 0.001) and those over 51 years of age (β = −0.381, *p* < 0.01), while a statistically significant difference was not observed in participants belonging to the age population of 41 to 50 years. A statistically significant effect of physical quality of work life (QWL-Ph) on quiet quitting was observed only in subjects under 40 years of age (β = −0.207, *p* < 0.01), while it was not observed in other age groups. Based on the results in Table 5, it was observed that in the relationships between QWL-P and QQ and QWL-Ph and QQ, both QWL-C and QQ were different in relation to the age of the subjects.

## 4. Discussion

This study aimed to investigate the relationship between the quality of work life and quiet quitting in healthcare professionals. The research focused on four dimensions of quality of work life: psychological, physical, social, and cultural quality of work life. In general, the results obtained identify a negative relationship between all observed dimensions of quality of work life and quiet quitting in healthcare professionals, which was also identified in one of the previous studies [31]. Although no studies have investigated the direct relationship between quality of work life and quiet quitting, several studies confirm that quality of work life is associated with variables related to the professional behavior of employees in a healthcare organization, such as productivity, absenteeism, fluctuation, conflicts with colleagues, and caregivers [34,61]. Studies also suggest that employee job dissatisfaction has a strong impact on increased turnover, employee disengagement, and a work environment in which tension prevails [4,19,62]. The results of our research and the above studies suggest that the quality of work life is an indicator that can have a major impact on the manifestation of quiet quitting in healthcare professionals. Job satisfaction is a variable that can be influenced by a positive work environment and positive emotional attitudes of employees towards work. For this reason, human resource managers have an extremely significant role in creating favorable working conditions and implement a human resource management strategy by providing support to healthcare professionals and thus stimulating positive emotions and desired behavior in employees.

The results of this study point to a negative relationship between the psychological quality of work life and quiet quitting healthcare professionals. The preservation of the mental health of healthcare professionals is becoming increasingly important, as it is recognized as a problem that can affect public health and the quality of health services provided [63]. The goal of any health organization is for working conditions to contribute to positive mental health, by developing a sense of well-being in the employee that leads to a positive experience that is manifested through happiness and satisfaction, which can contribute to employees being more productive in performing their work tasks [64]. However, healthcare professionals often feel pressure in the workplace due to the increased demand for increasingly complex health services, extended working hours, and the demand to provide the highest-quality services despite limited resources. In this way, co-workers are continuously exposed to stress, which can lead to burnout, depression, anxiety, sleep disorders, and other symptoms. Consequently, in employees who exhibit any of the above, the sense of well-being and subjective perception of the quality of life may be impaired, which can affect reduced productivity and lack of interest in the job. Studies show that psychological stress and burnout are associated with inadequate patient care, unprofessional employee behavior, and increased absenteeism from work [33,37,65]. All of this can have a negative impact on the well-being of employees and increase the possibility of quiet quitting, where employees perform minimum work, just enough to avoid being fired. It is crucial to identify and mitigate risk factors that impair the psychological quality of work life, to preserve the mental health and well-being of employees. Employees who have a positive attitude towards work have the support of their superiors [48] and quality sleep is characterized by high work performance and a lower turnover rate, all of which lead to better service delivery and greater customer satisfaction [65].

It is particularly important to point out that working conditions in healthcare organizations, such as high workload, length of working hours, standing for too long, and forced positions, can have a negative impact on the physical well-being of employees [66,67]. In our study, it was observed that the physical quality of work life has a negative and statistically significant impact on the manifestation of quiet quitting in healthcare professionals. Consequently, the health organization should adapt the working conditions to the employees and provide them with adequate support. The goal of occupational healthcare is to create a safe working environment for health professionals, which can prevent work from harming health that can negatively affect the professional behavior of employees. Employees who have a workplace that is in accordance with their physical abilities and the support of their superiors are less likely to feel fatigue and physical discomfort during work. When a work environment prevails in a healthcare organization where employees feel physically protected and safe, they can turn their attention to the dedicated provision of health services [68]. Consequently, employees can exhibit a higher level of physical quality of work life, which increases the likelihood that employees will be more dedicated to work and more productive and at the same time reduces the likelihood of healthcare professionals quietly quitting.

The next dimension of quality of work life examined is social well-being, and our results show a negative and statistically insignificant effect on quiet quitting. Although our results do not have statistical significance, they may be of interest for an additional analysis in future research.

In addition to the above, the level of quality of work life for employees is also influenced by cultural well-being, which refers to employees’ perception of the extent they can express their culture, traditions, and social norms. Some employees express their culture through productivity and individual choice, while others emphasize the importance of teamwork, free time, and work–life balance [69]. To meet these needs, it is extremely important that a positive organizational culture prevails in the health organization [70]. In our research, it was observed that the cultural quality of work life has a statistically significant and negative impact on the manifestation of quiet quitting in employees. Cultural dimensions are particularly important in shaping the behaviors, values, and attitudes of employees, including the way they perceive and manage work–life balance. When a culture that is focused on strengthening interpersonal relationships and respecting gender equality prevails in a healthcare organization, employees are more interested in their work, more productive, more likely to present innovative ideas, and more satisfied with their work. For employees who feel valued in the organization and have the support of their superiors, the possibility of quiet quitting is minimized [19].

The testing of multigroup effects found that there was a statistically significant effect in all dimensions of quality of work life on the manifestation of quiet quitting of healthcare professionals among men and women. On the other hand, healthcare professionals of all age groups were not statistically significant in terms of the impact of social quality of work life on the manifestation of quiet quitting, which confirms the previous conclusion that there is no statistically significant relationship between social quality of work life and quiet quitting. In healthcare professionals of both sexes, it was observed that the psychological quality of work life contributes to employees opting for quiet quitting, with a stronger effect observed in women. When looking at age, the impact of the psychological effect was observed in people over 40 years of age and those over 51 years of age. However, people over the age of forty are more likely to opt for quiet quitting when they have the perception that their psychological well-being has been impaired. Healthcare professionals under the age of forty are also more likely to opt for quiet quitting due to the perception that their work environment impairs their physical well-being. It is evident that the phenomenon of quiet quitting has a stronger impact on the younger generation, because in addition to demanding health services at the beginning of their professional careers, they also faced the COVID-19 pandemic [28]. During the pandemic, healthcare professionals faced additional stresses, including new safety procedures, wearing personal protective equipment, reduced workforce due to illness, overtime, lack of support, and rapid technological developments, all of which had an impact on their productivity and well-being [71]. Based on the above, it can be assumed that the experience and many years of continuous improvement of knowledge and skills in older healthcare professionals were crucial for them to feel safe and protected in the workplace and thus show a lower level of stress, and, at the same time, opt for behavior that leads to quiet quitting less often.

The relationship between social quality of work life and quiet quitting did not show statistically significant differences between employees in healthcare organizations by age, while a difference between sexes was observed, where this influence is more significant in the male population. According to Joseph and Tammy [72], men prioritize work and career development over family to provide them with financial stability, which may justify the importance of social quality of work life for men’s greater commitment to work. In terms of the impact of cultural quality of work life on quiet quitting, differences between women and men were observed, with a more significant effect in the female population. When looking at age, statistically significant results were identified in all age groups, with the greatest effects on healthcare workers aged 41 to 50 years. Healthcare professionals belonging to this age group are mostly on the rise in their professional careers. However, to maintain and increase their productivity, human resource managers should place special emphasis on satisfying the cultural quality of work life by promoting teamwork, strengthening interpersonal relationships, and reconciling work and private life. This can have a positive impact on organizational commitment, life and job satisfaction, stress reduction, and the intention to leave, all of which can contribute to a positive impact on employees not adopting the behavior of quiet quitting.

The results of this research have theoretical and practical implications. From the perspective of theoretical implications, the findings contribute to a better scientific understanding of the quality of work life and quiet quitting but also represent a valuable theoretical basis for future research. In practical terms, the results provide HR managers with insights into how an organization can develop an effective strategy for creating a positive work environment that fosters the satisfaction of quality of work life. In addition, the education of healthcare professionals is very important, because in order to cope with the challenges of the job, they need to have developed skills of moral resilience [48,73] and emotional intelligence [74], which can increase the possibility that the employee will not adopt quiet quitting [48].

## 5. Conclusions

The findings of this study lead to the conclusion that healthcare professionals’ perception of their quality of work life may have an impact on the manifestation of quiet quitting. In terms of quality of work life, cultural, psychological, and physical characteristics have had a significant negative impact on the behavior of employees in a health organization, especially among women. The importance of cultural well-being is more pronounced in healthcare professionals who are between 41 and 50 years old, while psychological and physical well-being is more significant in those under 40 years of age. By contrast, as important as social characteristics may seem to be for employee behavior, there was no significant impact on the manifestation of quiet quitting among healthcare professionals in all age groups, while social well-being was significant only for men. These results are significant because they provide insight into the dimensions of quality of work life indicators for the manifestation of quiet quitting in healthcare professionals.

Social exchange theory could explain relationships between quality of work life and quiet quitting by framing employment as an ongoing cost–benefit exchange. When healthcare professionals perceive that work demands (costs) outweigh rewards, such as a supportive climate, career prospects, and organizational commitment, the social deal loses value. This imbalance erodes employee well-being, heightens burnout, and fosters quiet quitting intentions [28]. Conversely, when the exchange is favorable, these same factors enhance quality of work life and sustain engagement, simultaneously preventing quiet quitting. Our study findings validate this exchange rationale: psychological, physical, and cultural quality of work life can negatively affect quiet quitting, while social quality of work exhibited no significant effect. The study’s findings are consistent with human need theory and demonstrate that unsatisfied needs in the domains of psychological, physical, and cultural quality of work life can lead to quit quitting [21].

The results of this study provide a basis for practical implications. In order to improve the quality of work life and mitigate quiet quitting risks, managers in healthcare organizations can consider several practical actions. First of all, healthcare organizations should cultivate a leadership style that combines transformational vision with day-to-day servant behaviors, including active listening, participation in decision-making, and modeling psychological safety, so that staff feel valued and heard. Special focus should be directed at implementing evidence-based talent strategies that attract and keep high-performing healthcare professionals. Practical steps in the realization of this strategy should include activities such as transparent career pathways, merit-based promotions, and flexible scheduling tied to life-stage needs. Embedding burnout management programs, which include regular workload reviews, access to confidential counseling, resilience workshops, and mandatory “micro-recovery” breaks, should ensure that support is tailored to individuals who need it most. Finally, interactional justice should be promoted by training managers through respectful feedback, enforcing zero tolerance for incivility, and institutionalizing open forums where employees can question decisions without fear of reprisal. The aforementioned managerial actions create the potential to elevate the quality of work life and stem the drift toward quiet quitting. In order to build and nurture employee well-being, human resource managers should focus their management actions on building a positive organizational culture, strengthening teamwork, providing adequate support to employees, assigning employees to workplaces that are in line with their personal characteristics, and implementing flexible work engagements with the aim of creating a work–life balance.

Given this study’s cross-sectional convenience sample design, the observed relationships are correlational only, and no causal inferences can be drawn. Considering the inherent limitations of using convenience sampling in our data collection process, future studies could strengthen representativeness by employing stratified or multi-stage cluster sampling across multiple healthcare institutions in the whole country to secure a more representative cohort of healthcare professionals. Our study does not prove a relationship between social quality of work life and quiet quitting, opening opportunities for additional research. Future studies should disaggregate the social quality of work life dimension (e.g., peer support, supervisor relations, teamwork climate) to clarify whether particular social facets exert context-specific influences on quiet quitting behavior.

## Figures and Tables

**Table 1:** Measurement model and constructs.

Construct and Items	Loadings	VIF	Cronbach’s Alpha	Composite Reliability	AVE
QWL-Ps: psychological quality of work life			0.891	0.891	0.755
QWL-Ps01	0.819	1.886			
QWL-Ps02	0.914	3.539			
QWL-Ps03	0.884	2.906			
QWL-Ps04	0.857	2.280			
QWL-Ph: physical quality of work life			0.853	0.871	0.691
QWL-Ph01	0.771	2.107			
QWL-Ph02	0.878	2.853			
QWL-Ph03	0.864	2.196			
QWL-Ph04	0.809	1.644			
QWL-S: social quality of work life			0.884	0.886	0.744
QWL-S01	0.839	2.091			
QWL-S02	0.903	3.641			
QWL-S03	0.902	3.520			
QWL-S04	0.802	1.724			
QWL-C: cultural quality of work life			0.922	0.922	0.810
QWL-C01	0.891	2.919			
QWL-C02	0.916	3.691			
QWL-C03	0.904	3.363			
QWL-C04	0.889	2.862			
QQ: Quiet quitting			0.869	0.876	0.524
QQ01	0.738	1.802			
QQ02	0.741	1.914			
QQ03	0.698	1.758			
QQ04	0.722	1.769			
QQ05	0.802	2.381			
QQ06	0.798	2.404			
QQ07	0.652	1.616			
QQ08	0.620	1.403			

**Table 2:** Discriminant validity (HTMT0.85 criterion).

Constructs	1	2	3	4	5
1. QWL-C: cultural quality of work life	–				
2. QWL-Ph: physical quality of work life	0.660				
3. QWL-Ps: psychological quality of work life	0.714	0.797			
4. QWL-S: social quality of work life	0.770	0.798	0.670		
5. QQ: quiet quitting	0.662	0.633	0.705	0.619	–

**Table 3:** Structural model fit indices.

Construct	Stoner–Geisser Q^2^	R^2^	GOF
Quiet quitting	0.462	0.474	0.467
SRMR	0.073		

**Table 4:** Results of testing direct effects.

Relationship	Path Coefficient	*t*-Value	95% CIs (Bias-Corrected)	Results
QWL-Ps → QQ	−0.307 ***	6.536	[−0.396, −0.212]	Supported
QWL-Ph → QQ	−0.136 **	2.750	[−0.230, −0.037]	Supported
QWL-S → QQ	−0.100	1.911	[−0.206, 0.002]	Not supported
QWL-C → QQ	−0.247 ***	5.688	[−0.330, −0.162]	Supported

Notes: QWL-Ps, psychological quality of work life; QWL-Ph, physical quality of work life; QWL-S, social quality of work life; QWL-C, cultural quality of work life; QQ, quiet quitting. ** *p* ˂ 0.01; *** *p* ˂ 0.001.

**Table 5:** Results of testing direct effects: multigroup partial least square path modeling.

Relationship	Path Coefficient	*p*-Value	Path Coefficient	*p*-Value	Path Coefficient	*p*-Value	Invariant
	Female	Female	Male	Male			
QWL-Ps → QQ	−0.308	0.000 ***	−0.297	0.015 *			Yes
QWL-Ph → QQ	−0.174	0.001 **	−0.005	0.963			No
QWL-S → QQ	−0.057	0.324	−0.295	0.001 **			No
QWL-C → QQ	−0.233	0.000 ***	−0.299	0.004 **			Yes
	Age < 40	Age < 40	Age 41–50	Age 41–50	Age > 51	Age > 51	
QWL-Ps → QQ	−0.336	0.000 ***	−0.152	0.152	−0.381	0.001 **	No
QWL-Ph → QQ	−0.207	0.003 **	−0.161	0.070	−0.086	0.461	No
QWL-S → QQ	−0.039	0.603	−0.174	0.084	−0.103	0.295	Yes
QWL-C → QQ	−0.226	0.001 **	−0.322	0.000 ***	−0.187	0.031 *	Yes

Notes: QWL-Ps, psychological quality of work life; QWL-Ph, physical quality of work life; QWL-S, social quality of work life; QWL-C, cultural quality of work life; QQ, quiet quitting. * *p* ˂ 0.05; ** *p* ˂ 0.01; *** *p* ˂ 0.001.

## Data Availability

The data presented in this study are available on request from the corresponding author due to ethical reasons.
